# Control of human trophoblast function

**DOI:** 10.1186/1477-7827-5-6

**Published:** 2007-02-08

**Authors:** Laura Lunghi, Maria E Ferretti, Silvia Medici, Carla Biondi, Fortunato Vesce

**Affiliations:** 1 Department of Biology, Section of General Physiology, University of Ferrara, 44100 Ferrara, Italy; 2 Department of Biomedical Sciences and Advanced Therapy, Section of Obstetrics and Gynaecology, University of Ferrara, 44100 Ferrara, Italy

## Abstract

The trophoblast, i.e. the peripheral part of the human conceptus, exerts a crucial role in implantation and placentation. Both processes properly occur as a consequence of an intimate dialogue between fetal and maternal tissues, fulfilled by membrane ligands and receptors, as well as by hormone and local factor release. During blastocyst implantation, generation of distinct trophoblast cell types begins, namely the villous and the extravillous trophoblast, the former of which is devoted to fetal-maternal exchanges and the latter binds the placental body to the uterine wall. Physiological placentation is characterized by the invasion of the uterine spiral arteries by extravillous trophoblast cells arising from anchoring villi. Due to this invasion, the arterial structure is replaced by amorphous fibrinoid material and endovascular trophoblastic cells. This transformation establishes a low-resistance, high-capacity perfusion system from the radial arteries to the intervillous space, in which the villous tree is embedded. The physiology of pregnancy depends upon the orderly progress of structural and functional changes of villous and extravillous trophoblast, whereas a derangement of such processes can lead to different types of complications of varying degrees of gravity, including possible pregnancy loss and maternal life-threatening diseases. In this review we describe the mechanisms which regulate trophoblast differentiation, proliferation, migration and invasiveness, and the alterations in these mechanisms which lead to pathological conditions. Furthermore, based on the growing evidence that proper inflammatory changes and oxidative balance are needed for successful gestation, we explain the mechanisms by which agents able to influence such processes may be useful in the prevention and treatment of pregnancy disorders.

## Introduction

Trophoblast is an embryonic tissue which exerts a crucial role during implantation and placentation. Both processes can only take place through a significant change in the uterine wall in response to different modulatory molecules, among which steroid and peptide hormones, as well as local factors including prostanoids. This transformation, aimed at creating a favourable environment for receiving the blastocyst, and permitting embryo-fetal development, is represented by a complex series of events termed decidualization. Implantation consists of the blastocyst penetrating the luminal epithelium, crossing the basal lamina and, finally, embedding itself in the stroma. During implantation a syncytiotrophoblast (ST) is formed, which begins to invade the maternal tissue. Afterwards, vascularization of the trophoblast occurs in order to establish and maintain a feto-placental vasculature. Simultaneously, maternal vascular remodeling takes place so as to generate a utero-placental circulation. For successful placentation to occur, a highly orchestrated control of vasculogenesis, angiogenesis, and trophoblast functions is required. This is operated by a large number of heterogeneous factors which act by both autocrine and paracrine mechanisms.

Here we review the control of trophoblast function, highlighting the aspects which may improve management of pregnancy complications.

## Decidualization

In order for implantation to occur, endometrium has to be changed into decidua. This process consists in modifying endometrial stromal cells, uterine glands and vessels, as well as the population of uterine immune cells. In humans, unlike other species [1], decidualization is independent of the blastocyst's presence in the uterine cavity and begins in the late secretory phase of the menstrual cycle. It is evoked by progesterone, as well as by regulatory agents able to enhance cyclic AMP (cAMP) levels [2,3]. Decidualization continues in pregnancy, and it is thought to regulate subsequent trophoblast invasion and placenta formation by altering the expression of regulatory factors such as metalloproteinases, cytokines, surface integrins, and major histocompatibility complex molecules. The trophoblast, in turn, releases paracrine signals which modulate decidual stromal cell gene expression [4]. These cells become round and show ultrastructural similarities with myofibroblasts and epithelial cells [5]. Moreover, they release several factors including prolactin, relaxin, renin, insulin-like growth factor binding protein-1 (IGFBP-1) [1,3] and specific extracellular matrix (ECM) proteins such as laminin and fibronectin [6]. *In vitro *studies have demonstrated that this event is correlated with changes in steroid hormone receptor expression and steroid metabolism, remodeling of the ECM and cytoskeleton, altered expression of enzymes, growth factors and cytokines, and induction of apoptosis modulators and specific transcription factors [7]. Elongation of the spiral arteries occurs through an endometrium-specific angiogenesis, characterized by proliferation of both endothelial and smooth muscle cells, with preservation of the integral structure of the vessel. In the uterine wall, several leukocyte types, such as T lymphocytes, a few B lymphocytes, macrophages, and natural killer (NK) cells, are present. However, after ovulation, a dramatic increase in NK cells is observed. Uterine NK cells (uNK) are phenotypically and functionally different from circulating ones. Indeed, they have not cytolytic activity [8], and express integrins which allow their migration and invasion of the decidualizing endometrium [6]. It has been suggested that the unique environment resulting from the transformation of endometrium to decidua plays a crucial role in uNK cell specificity acquisition [6,9]. Since decidual NK cells decrease in number in the second half of pregnancy and disappear at delivery, it has been hypothesized that their main role is confined to early pregnancy, when they modulate implantation and placentation interacting with both decidual stromal cells and trophoblast [6,9]. Interestingly, it has been shown that uNK cell recruitment is a hormonally-controlled maternal function and is independent of the presence of the implanting embryo [10]. NK cell survival seems to be dependent upon the presence of progesterone, whose action, however, is presumably not direct, but mediated by decidual stromal cells which express hormone receptors [11]. In contrast, it has recently been shown that glucorticoids are able to decrease the number of decidual NK cells [12].

Decidualization is also characterized by a decrease in Th1 and an increase in Th2 lymphocytes, an effect which is evoked by progesterone and cytokines. It has been proposed that Th2 cytokines protect fetus and trophoblasts inhibiting NK cell cytotoxicity and proliferation, shifting NK cell cytokine production toward a Th2 phenotype, as well as suppressing cytotoxic T cells activation [12-14].

## Blastocyst implantation

An intimate cross-talk between the embryo and the uterus is needed for blastocyst implantation [4,15]. This process, which consists of an interaction between trophoblast cells and endometrium, can only take place in a restricted period of time, termed "window of receptivity". It is initially dependent upon the presence of estrogen and progesterone, although further morphological and biochemical changes are evoked within the uterine wall by signals from the embryo and invading trophoblast. The "window of receptivity" in humans is presumed to span days 20–24 of the menstrual cycle [1]. Indeed, out of this period the epithelium apical surface is covered by a thick glycocalyx, mainly composed of mucin, and in particular MUC1, a transmembrane glycoprotein characterized by an extended extracellular domain which prevents blastocyst attachment [16]. Blastocyst implantation is also impaired by the large number of desmosomes which exist along lateral epithelial cell surfaces [17]. In some species the "window of receptivity" is characterized by down-regulation of MUC1, but this condition has not been observed in humans. Some researchers have suggested that MUC1 may actually promote human blastocyst attachment to the uterine wall [18], whereas others demonstrated a loss of this mucin at the site of blastocyst interaction [19] thanks to uterine proteases, activated by factors released from the blastocyst itself [16].

Blastocyst attachment to the uterine wall depends upon the interaction between adhesion molecules such as selectins, integrins, and trophinins [17], expressed on both trophoblast cells and uterine epithelium. This interaction is mediated, in most cases, by bridging ligands including, at least in the sheep, osteopontin and galectin-15, which are released in the uterine cavity by endometrial glands [20]. Invasion is favoured by the simultaneous decrease of desmosome density and basal membrane digestion, finally leading to nidation in the decidual stroma [17].

A key role in the control of human blastocyst implantation is exerted by endometrial chemokines and cytokines. Chemokines are thought to be responsible for the promotion of leukocyte migration to the decidua, where they cause a sort of inflammatory state, a process which appears to derive from several sources. First, decidualization is characterized by NK cells interaction with the non-polymorphic HLA class I antigens expressed by invading trophoblasts [21]. Furthermore, signalling agents secreted by seminal vesicles and prostate gland interact with epithelial cells in the cervix and uterus, recruiting and activating macrophages, granulocytes and dendritic cells. They are provided with immune-regulatory and tissue remodelling roles that improve endometrial receptivity to the implanting embryo. Tumor growth factor β (TGF-β) and prostaglandins (PGs) present in seminal fluid contribute to enhance cytokine production and vascular permeability, that appear to be essential for implantation, due to their effects favouring blastocyst attraction and attachment to the endometrium [13]. Moreover chemokines, interacting with G protein-coupled receptors, induce a structural change in integrins which favours adhesion of the blastocyst to the decidualized endometrium [22]. NO favours blastocyst implantation in both animals and humans, modulating PG release, ovarian steroidogenesis, uterine cell proliferation, glandular secretion and blood flow, as well as mediating sex steroid and growth factor actions [15]. Since normal pregnancy is a physiological process, in our opinion it should not be defined as a 'controlled state of inflammation' [23]. However, at an early stage at the implantation site, as well as later systemically, it is regulated by the same cytokines whose derangement can trigger the inflammatory pathway, leading to various types of early and late gestational diseases. Under such profile, the above mentioned 'Th1/Th2' shift hypothesis, that dominated reproductive immunology for many years, should be more simply interpreted as the expression of the modulation of the cytokines that regulate the vascular processes of placentation, rather than a specific mechanism to avoid fetal T-cell rejection.

## Trophoblast differentiation and function

The formation of floating and anchoring villi begins immediately after nidation. Three stages are recognized in the development of the villi, namely primary, secondary and tertiary villi, the last being characterized by a de-novo formation of capillaries from mesenchymal precursors and thus devoted to feto-maternal exchanges. Villous tree expansion occurs throughtout pregnancy [24].

The specialized villous cell types, i.e. ST and extravillous trophoblast (EVT) cells, originate from cytotrophoblast (CT) stem cells. During blastocyst implantation, CT cells fuse to form an external layer of non-proliferative multinucleated ST which then grows thanks to the steady incorporation of new CT cells. ST exerts a crucial role in feto-maternal exchanges and possesses endocrine activity, releasing hormones involved in the homeostasis of pregnancy such as chorionic gonadotrophin (CG) and placental lactogen (PL). Around day 14 after implantation, CT cells break through the ST layer giving rise to EVT cells, which begin to invade the uterine stroma as trophoblastic cell columns. From the tips of the columns, a subpopulation of these motile and invasive cells moves laterally, to form the trophoblastic shell, and longitudinally to invade deeply the decidua and the deeper portion of the myometrium, where these cells evoke profound changes within the uterine vessels [25].

## Factors involved in the control of CT differentiation

Differentiation of CT to ST or EVT cells is precisely controlled by different agents such as transcription factors, specific genes, hormones, growth factors, cytokines and O_2 _levels. The glial cell missing factor 1 (GCM1), together with the AP-2 and Sp transcription factor families, stimulate syncytial fusion, whereas Hash-2 inhibits this process by reducing CYP19/aromatase gene transcription [26]. Hash-2 and Id-2 maintain CT proliferation, and their downregulation is observed in differentiated cells [27,28]. The expression of some of the above mentioned transcription factors is influenced by O_2 _levels and cAMP.

A relatively low oxygen environment characterizes blastocyst implantation, placentation and early embryonic development up to the 10th week of gestation [29]. Accordingly, in the first trimester, the villous trophoblastic layer is twice the thickness it becomes in the second. Moreover, the intervillous circulation is established peripherically at around the 9^th ^week, and it enlarges to encompass the whole placenta only after the 12^th ^week. This could be due to the presence of trophoblast cell plugs which occlude the tips of the uteroplacental arteries [30], or to incomplete spiral artery remodeling [31]. Low O_2 _levels stimulate CT proliferation and inhibit EVT and ST differentiation. Hypoxia acts by modifying gene expression or mRNA stability [32]. For instance it stimulates the expression of both the hypoxia inducible factor-1 (HIF-1), which maintains CT proliferation, and Hash-2, thus preventing differentiation. Moreover it has been reported that, in first-trimester trophoblast, HIF-1 expression parallels that of TGF-β3, an inhibitor of early trophoblast differentiation which impairs its acquisition of the invasive phenotype [31,33]. In line with this last report, it has been observed that invading CT downregulates the expression of both HIF-1 and TGF-β3 genes [33,34]. cAMP also seems to be involved in trophoblast differentiation and it has been shown to influence ST formation. It enhances AP-2 and Sp functionality, promotes the expression of the syncytin gene, a highly fusogenic membrane protein localized at the CT-ST interface, and mediates the syncytialization evoked by CG [35]. Among growth factors, the vascular endothelial growth factor (VEGF), whose expression has been demonstrated in decidual cells, CT and EVT [36-38], stimulates CT differentiation to both ST and endovascular EVT, and its effect is inhibited by soluble fms-like tyrosine kinase-1 (sFlt-1), which acts by binding VEGF [39].

**Figure 1:**
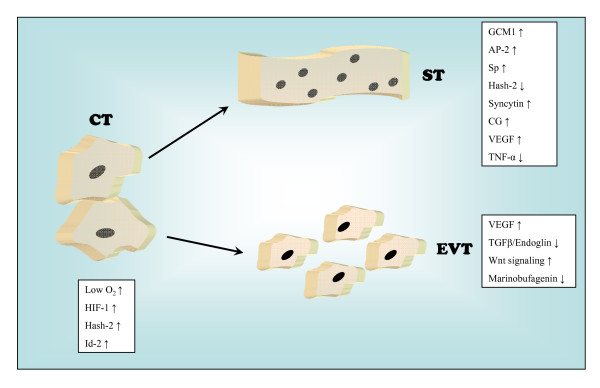
Schematic representation of cytotrophoblast (CT) differentiation to syncytiotrophoblast (ST) or extravillous trophoblast (EVT), together with some of the key factors involved in the control of these events. The box on the left lists factors stimulating CT proliferation, thus inhibiting differentiation. Boxes on the right list factors stimulating or inhibiting ST or EVT cell formation.

TGF-β, instead, inhibits differentiation along the invasive pathway helped by endoglin, a component of TGF-β receptor complex [40], while tumor necrosis factor (TNF)-α reduces syncytialization [41]. The involvement of canonical Wingless (Wnt) signaling and of marinobufagenin, the endogenous sodium pump inhibitor, has recently been reported in the acquirement of the invasive phenotype [42,43]. An alteration of trophoblast differentiation may result in pregnancy diseases, such as preeclampsia. For instance a persistent hypoxia, together with an increase of HIF-1 and TNF-α as [44] well as a decrease of syncytin expression [45], induces a decrement of syncytialization and consequently an enhancememnt of trophoblast apoptosis, an event notoriously involved in preeclampsia pathogenesis. A schematic representation of the above described control of trophoblast differentiation is reported in Fig. 1.

## Extravillous trophoblast function

**Figure 2:**
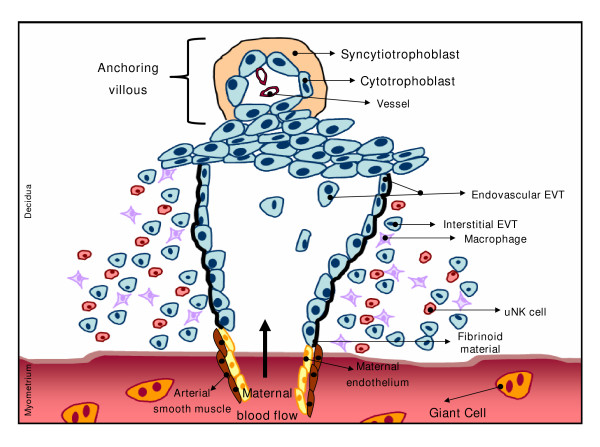
Maternal spiral artery remodeling through the combined action of interstitial and endovascular extravillous trophoblast cells.

EVT cells originating from the trophoblastic shell first enter the decidua and subsequently the myometrial stroma [3] as interstitial trophoblast. This encircles and destroys the smooth muscle cells of spiral artery media which is replaced by amorphous fibrinoid material. Subsequently, EVT expressing an endothelial phenotype invade the lumen of the arteries [46] to replace the endothelium of the vessels (Fig. 2).

Invasion of endometrial vessels by endovascular EVT is evident from 8 weeks onward, whereas myometrial artery invasion begins around the 14^th ^week. This process mainly involves the vessels in the center of the placental bed, but also to a lesser extent the peripheral vasculature [47]. The expression of both angiopoietins and their receptor (Tie-2) has been observed during early placentation, suggesting an involvement of these regulatory agents in vascular remodeling. Moreover, the demonstration that Tie-2 is expressed on trophoblast suggests that angiopoietins may regulate its functions. For instance, it has been demonstrated that angiopoietins stimulate proliferation and migration of cultured cytotrophoblast and EVT cells, respectively [36]. Since the guinea pig interstitial trophoblast expresses NO synthases [48], a role for NO in dilating and remodeling uterine vessels has been hypothesized. However, human invasive trophoblast does not express NO synthesizing enzymes [49], and therefore it should not be able to release this vasoactive molecule. Another putative vasodilator is carbon monoxide, produced by hemoxygenase, whose expression has been demonstrated in all EVT cells [50]. Furthermore, molecules able to interact with each other have been identified in both NK and EVT cells [9]. In EVT cells MHC class I molecules are expressed, in particular HLA-C, E and G that are all good ligands for several members of the killer immunoglobulin receptor (KIR) family, present on NK cells. Such interaction modifies the NK cell cytokine repertoire and regulates adhesion molecules as well as matrix metalloproteinase (MMP) functionality [51]. Interstitial EVT cells move to the inner myometrium, where they fuse to become placental bed giant cells (GC) [52]. Since these multinuclear cells lose their ability to migrate and invade, their formation is likely to represent a mechanism which prevents deeper penetration into the uterine wall.

Invading EVT cells up-regulate the expression of proteins which favour uterine wall invasion, including MMPs, α5β1 and α1β1 integrins, VE-cadherin, and the trophoblast specific HLA class 1 molecule (HLA-G) which probably exerts a role in preventing fetal rejection. Conversely, these cells down-regulate the expression of adhesion molecules, such as α6β4 integrin or E-cadherin, unqualified for the invasion process, or of regulatory factors which inhibit cell invasiveness [53].

In addition to CT differentiation, O_2 _levels also influence EVT cell function. An inhibitory effect of hypoxia on EVT cell invasiveness has been reported [31,33,54], which is thought to be due to a modification of the integrin expression pattern which is, in turn, influenced by components of the ECM [54]. However, under different experimental conditions, an enhancement of trophoblast cell line invasiveness has also been observed [55]. This effect has been related to an enhanced expression of urokinase-type plasminogen activator (uPAR), an event which then results in the activation of plasmin and latent MMPs [55]. According to Genbacev *et al *[31] hypoxia does not inhibit cytotrophoblast differentiation/invasion before the 7^th ^week of gestation.

Modulation of EVT function is a complex phenomenon which depends on a growing number of factors [13,56], beside those above described. However, the majority of available data were obtained from *in vitro *experiments, and contrasting responses may derive from different experimental conditions [57,58]. It is therefore impossible at present to describe the *in vivo *picture. Some of the key regulators of EVT functions are listed in Table 1.

**Table 1:** some of the key factors regulating EVT cell functions

**Factors**	**Source**	**Effect**			**Ref.**
			
		**Proliferation**	**Migration**	**Invasiveness**	
**Adhesion molecules**	Trophoblast		↑↓	↑↓	53
**Angiopoietins**	Decidua, trophoblast		↑		36
**Colony Stimulating Factor-1**	Placenta, decidua	↑			59
**Decorin**	Decidua	↓	↓	↓	60
**Epidermal Growth Factor**	Decidua, trophoblast	↑		↑	61
**Endothelin**	Placental blood vessels, CT, ST, EVT		↑		62
**Hepatocyte Growth Factor**	Decidua, trophoblast		↑	↑	63
**Insulin-like Growth Factor II**	Trophoblast		↑	↑	64,65
**Insulin-like Growth Factor Binding Protein-1**	Decidua		↑	↑	64,65
**Melanoma Cell Adhesion Molecule**	Uterine smooth muscle cells		↓		66
**Metalloproteinases**	Trophoblast			↑	67
**Nodal**	Placenta	↓			68
**Hypoxia**				↑↓	54,55
**Urokinase-type Plasminogen Activator**	Trophoblast		↑	↑	69
**Prostaglandin E_2_**	Decidua, trophoblast		↑		57
		↓	↓		58
**8-iso-PGF_2α_**	Decidua			↓	70
**Placental Growth Factor**	Trophoblast	↑			71
**TGF-β**	Decidua, trophoblast, uNK cells	↓	↓	↓	61,64
**TNF-α**	Decidua, uNK cells, decidual macrophages		↓		72
**VEGF**	Decidua, trophoblast	↑			73

## The influence of the oxidative balance on trophoblast functions and embryo development

The oxidative balance in the gestational sac, besides influencing CT differentiation and EVT cell function, affects embryo survival and development. It is known that hypoxia in embryonic stem cells evokes HIF-1 gene expression which, in turn, activates multiple genes involved in both proliferation/differentiation and in cellular adaptive responses to low O_2 _availability [74].

**Figure 3:**
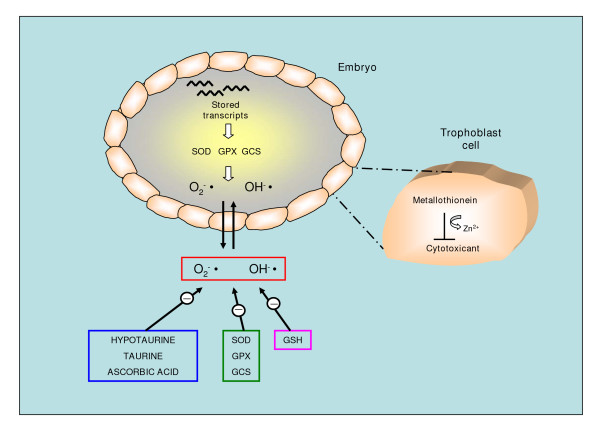
Antioxidant mechanisms which protect the embryo against ROS excessive formation. Superoxide dismutase (SOD), glutathione peroxidase (GPX) and gamma-glutamylcysteine synthase (GCS) transcripts have been identified in the oocyte, embryo and oviduct. The non-enzymatic antioxidants hypotaurine, taurine and ascorbic acid have been found in follicular and tubal fluids. Metallothionein is synthesized by trophoblast cells, and acts by neutralizing ROS and releasing the cell survival factor Zn^2+^.

The hypoxic milieu, in which the conceptus develops in early pregnancy, may serve to protect fetal tissues and developmental processes against the deleterious effects of the reactive oxygen species (ROS), during critical phases of embryogenesis and organogenesis. This protection is potentiated by the existence of internal and external antioxidant defense mechanisms. The former mainly comprise antioxidant enzymes such as superoxide dismutase, glutathione peroxidase and gamma-glutamylcysteine synthase. Transcripts encoding for these enzymes have been demonstrated in the oocyte, embryo and oviduct, and their expression seems essential to embryo acquisition of the propensity to develop. External defense mechanisms are represented by non-enzymatic antioxidants such as hypotaurine, taurine and ascorbic acid which are found in follicular and tubal fluids [75]. On the other hand, it has been established that human trophoblast cells, exposed to ROS or other cytotoxicants, overexpress the metal-binding protein metallothionein, which exerts a protective role by both direct and indirect mechanisms. In fact this protein is thought to bind and hence neutralize these toxicants, simultaneously releasing normally bound zinc, which possesses cytoprotective actions [76]. A schematic representation of defence mechanisms against ROS is reported in Fig. 3.

O_2 _tension increases sharply at the end of the first trimester, when an enhancement of oxidative stress markers as well as of mRNAs for antioxidant enzymes occurs in trophoblast cells [77]. Such enzyme expression could be induced by NAD(P)H oxidases [78], stimulated by hypoxia itself, which leads to intracellular superoxide anion production. This subsequently upregulates the expression of antioxidant enzymes, possibly contributing to modulation of EVT function. Indeed, it has been demonstrated that superoxide evokes the expression of HIF-1 and other genes related to vascular and matrix remodeling, as well as to cell apoptosis [78]. On the other hand, it is believed that a premature and widespread onset of maternal placental circulation, before the development of antioxidant defense in the placenta, is correlated to oxidative trophoblast damage and could represent a cause of early pregnancy loss [30].

The demonstration that physiological concentrations of ROS exert beneficial effects has led to the formulation of the "free radical theory of development". According to this theory, time course and pattern formation in developing organisms may be influenced by differential O_2 _supplies and/or gradients in the intracellular redox state [79]. Indeed, it is known that low ROS concentrations stimulate cell proliferation as well as gene expression, and it has been suggested that ROS favourable actions may be related to their control of the local inflammatory reaction. Recently, it has been evinced that physiological levels of ROS induce the expression of several cellular transcriptional factors, through the activation of multiple signal transduction pathways [80]. In contrast, high ROS levels are notoriously detrimental for cell survival and functions. *In vitro *investigations showed that the increased O_2 _requirement by developing embryos enhances the rate of ROS production, thus representing a potential teratological threat to fetal tissues, as shown in the mouse model [81]. Moreover, injury caused by oxidative stress may be responsible for developmental retardation and arrest of mammalian preimplantation embryos. As for the mechanism responsible for this, it has been hypothesized that high ROS formation may initiate and propagate an inflammatory process, resulting in placental tissue apoptosis [82].

The role and modulation of the oxidative balance in pregnancy have recently been reviewed by Biondi *et al *[83].

## Pathologic pregnancy

Perturbation of trophoblast functions may result in a range of adverse pregnancy outcomes such as malformation, fetal growth retardation, spontaneous abortion and stillbirth. For instance, a limited trophoblast invasion of maternal vessels has been correlated to both preeclampsia and fetal growth restriction, whereas an excessive trophoblast invasion is associated with invasive mole, placenta accreta and choriocarcinoma.

However, in spite of the the quantity of literature regarding the physiopathology of trophoblastic functions, the mechanisms leading to a successful pregnancy are far from fully understood. A modern approach to the matter is represented by the attempt to investigate the behaviour of the regulatory factors which are known to carry a high risk of spontaneous abortion in pregnancies, such as those complicated by fetal aneuploidy. Results obtained in this field demonstrate that chromosomal alterations in the embryo are correlated with anomalous amniotic and maternal plasma levels of growth factors and proinflammatory cytokines [84,85] which may impair trophoblast function. This concept is supported by the demonstration that trisomy 21 is associated with various defects in CT differentiation represented by down-regulation of adhesion molecules such as integrin α1 and, possibly as a compensatory mechanism, upregulation of MMP-9. These alterations may be responsible for the increase in CT apoptosis at the maternal-fetal interface [86].

Preeclampsia, a multifactorial syndrome thought to be caused by a combination of genetic, environmental, immunological and nutritional factors affects approximately 2–3% of all pregnant women and is a major cause of maternal and fetal morbidity and mortality. It is generally diagnosed in the third trimester and it is frequently, though not necessarily, responsible for pregnancy-induced hypertension and proteinuria. The pathological basis behind the clinical symptoms is represented by generalized vasoconstriction, increased vascular reactivity, parenchymal hypoperfusion, excessive edema, and platelet activation triggering the coagulation cascade [87].

**Figure 4:**
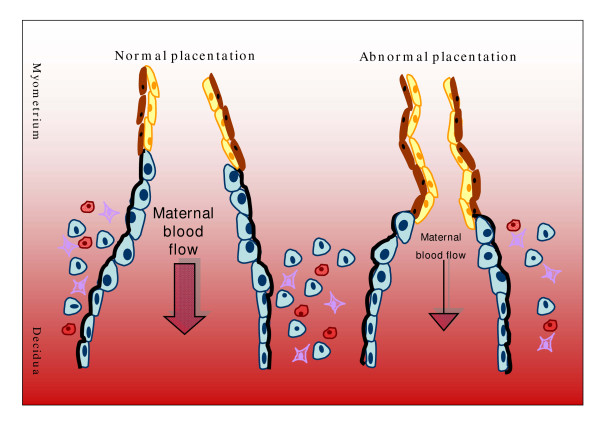
Spiral artery remodeling in normal and abnormal placentation. During normal placentation, EVT cells invade uterine wall and maternal spiral arteries replacing smooth muscle with fibrinoid material and part of vessel endothelium, thus evoking artery dilatation. Decidual immune cells, like macrophages and NK cells, facilitate deep invasion of EVT cells up to myometrial portions of spiral arteries. A limited EVT cell invasion, in abnormal placentation, impairs the formation of the high-capacity, low-resistance feto-maternal circulation needed for an adequate oxygen and nutrient supply for the growing fetus. For cell type description, see figure 2.

In normal pregnancy, as described above, EVT cells transform the spiral arteries into low-resistance vessels. In preeclampsia, however, spiral artery remodeling is defective and the utero-placental circulation remains in a state of high resistance (Fig. 4). It has been hypothesized that poor placental perfusion *per se *is an insufficient prerequisite for preeclampsia; as a matter of fact, this pathologic condition appears only when altered placentation occurs together with maternal constitutional factors [69]. Both the mother and fetus contribute to preeclampsia, the fetal contribution being affected by paternal genes. Indeed, Hiby *et al *[88] recently indicated the following factors correlated to preeclampsia: HLA-C, on fetal trophoblast cells and KIRs, on maternal decidual NK cells. Both of these factors are characterized by an extensive polymorphism of immunological importance in this condition. In particular they suggest that mothers lacking most or all activating KIR are at a greatly increased risk of preeclampsia when the fetus possesses HLA-C belonging to the HLA-C2 group. In preeclampsia the dialogue between EVT and NK cells necessary for a correct spiral artery remodeling during early pregnancy cannot take place.

Several other factors have been implicated in the poor remodeling of spiral arteries, such as a defect in EVT cell differentiation toward the invasive phenotype, an increase in apoptosis, an imbalanced control of migratory and invasive EVT functions, and the inability of cells to adopt an endovascular phenotype [69,89,90]. In contrast, Brosens *et al *[3] proposed the scarce myometrial artery transformation may be due to a deficient myometrial decidualization, rather than to defective trophoblast invasion. Nevertheless, it has been reported that in preeclampsia CT cells fail to down-regulate α6β4, to up-regulate α1β1 integrins and to enhance MMP and HLA-G expression, whereas they maintain an elevated production of E-cadherin and of the anti-invasive factor, TGF-β [52]. Moreover, Redline *et al *[91] demonstrated that this pathological condition is associated with an excess of proliferative immature intermediate trophoblast cells, probably due to dysregulation of some cytokine and growth factor secretion. Indeed altered levels of several cytokines, produced at the maternal-fetal interface and involved in the physiological control of EVT cell proliferation, differentiation and function, have been found in the blood of preeclamptic women [87]. It has also been hypothesized that enhancement of IL-2 production, due to the reduced placental HLA-G expression, is responsible for the scarce invasiveness of preeclamptic trophoblast [92], and that deficiency of IL-10 may contribute to enhanced inflammatory responses elicited by TNF-α and interferon-γ towards the trophoblast [50]. The enhanced pro-inflammatory/anti-inflammatory cytokine ratio is mainly due to the shift to a Th1-predominant state, clearly demonstrated in preeclampsia, and probably associated to an excessive production of inflammatory agents, among which IL-12 [14]. Abnormalities in TGF-β3 expression are also associated with preeclampsia and it has been demonstrated that down-regulation of this growth factor restores the invasive capability of preeclamptic trophoblast cells [34]. Caniggia *et al *[33] hypothesized that an up-regulation of both TGF-β3 and HIF-1 expression, secondary to a failure in the change in O_2 _tension during early placentation or to a defect in the ability of trophoblast cells to respond to this change, could arrest trophoblast differentiation along the invasive pathway. Alterations in the invasion-regulating system, uPA/uPAR/PAI, may also contribute to the development of preeclampsia since reduced levels of uPA and increased concentrations of PAI-1 have been reported in preeclamptic mothers. Such an observation is in line with the demonstration that most of the MMP-9 secreted by the preeclamptic trophoblast is in an inactive form, and that antibodies against uPAR are found in pregnant women with a history of fetal loss [69].

Placental ischemia, consequent to poor spiral artery remodeling [87], enhanced Th1/Th2 ratio [93] and pro-angiogenic/anti-angiogenic factor imbalance [94] may promote inflammatory changes through the release of Th-1 cytokines and ROS with consequent endothelial dysfunction, leading to the release of humoral factors responsible for the clinical symptoms of preeclampsia [69].

Several pieces of evidence suggest an involvement of ROS in endothelial alterations of the syndrome [69,83,95,96]. High decidual levels of oxidative stress markers have been found in preeclamptic decidua [95], and some of these are able to inhibit EVT cell invasiveness [96]. Moreover a reduction in glutathione peroxidase has been demonstrated in the preeclamptic placenta, probably correlated with increased *in vitro *placental production of lipid hydroperoxides and thromboxane A_2 _(TXA_2_), a vasoconstrictive and pro-aggregatory compound, normally counterregulated by prostacyclin (PGI_2_). The consequent TXA_2_/PGI_2 _imbalance could contribute to the state of high resistance of the utero-placental circulation in preeclampsia. Since NO enhances vasodilatatory action of PGI_2 _and inhibits TXA_2_-mediated-vasoconstriction, its reported decrease in preeclampsia could worsen vascular dysfunction [97]. An enhancement of placental NAD(P)H oxidase activity, possibly stimulated by the increased vascular resistance, has recently been implicated in preeclampsia. Excessive superoxide production could be detrimental, both directly and indirectly, through an increase of cytokine expression [77].

It has been reported that trophoblast cell-derived debris, a by-product of apoptosis in the outer layers of the developing and mature placenta, is present in maternal blood during normal pregnancy. It increases in the blood of preeclamptic women, probably due to an exaggerated apoptosis or even ischaemic necrosis of the oxidatively stressed placenta. This event could represent a further pathogenic mechanism of preeclampsia, through the release of pro-inflammatory cytokines [98].

## Therapeutic approaches

In spite of a large amount of research in recent years, the mechanisms underlying physiological pregnancy and their changes which trigger pathological conditions are not completely understood. However, a strong correlation between the features of the inflammatory response and the adverse outcomes of pregnancy has recently been found. Indeed, inflammation is able to subvert the physiological changes that regulate placental perfusion and myometrial tone. Further investigation into the molecules involved in physiological and pathological pregnancy should pave the way to their use, as well as that of their agonists and antagonists, as therapeutic agents. From this perspective, control of the maternal inflammatory response and its influence on vascular and myometrial functions can be accomplished by administration of progesterone and glucocorticoids. Indeed, it has already been established that these hormones influence Th1/Th2 balance in early pregnancy by inhibiting Th1 response, thus favouring anti-inflammatory cytokine production [99,100]. It is also known that, in cases of recurrent pregnancy loss, a Th1 milieu which increases peripheral NK cell number and infiltration of such cells in the endometrium is formed [12,101]. Furthermore, it has recently been demonstrated that glucocorticoids at doses equivalent to those which enhance fetal lung maturity inhibit pro-inflammatory cytokine production in explants from normal and preeclamptic placenta, without altering anti-inflammatory cytokine release [102]. In addition, it has been observed that, at least in mice, progesterone action is exerted through an interaction with glucocorticoid receptors and that corticosterone is 10–100 times more effective than progesterone [99]. However, while progesterone is currently employed as a therapeutic tool during early gestation, the use of glucocorticoids is generally still restricted to the prevention of neonatal respiratory distress syndrome, necrotizing enterocolitis and other severe third trimester complications. The reasons for such a clinical attitude are not clear. They may be due to a deeper concern about the teratogenic effects of glucocorticoids compared with progesterone. Corticosteroid treatment has been found to be ineffective in recurrent pregnancy loss associated with autoantibodies [103]. However it has been reported that low doses of glucocorticoids are not only harmless but even effective in the protection of pregnancy in cases of recurrent abortion of unknown origin [104]. Such efficacy is confirmed daily in our clinical experience. Moreover, recent reports indicate a beneficial effect of preconceptual prednisolone treatment on the outcome of pregnancy in women with history of unexplained recurrent abortion and characterized by elevated levels of NK cells within the endometrium before treatment. In all of these cases, NK cells are reduced upon prednisolone administration [105-107]. Knowing that uNK recruitment is a hormonally-controlled maternal function [10], and that an increased number of these cells is associated with recurrent miscarriage, NK cell cytotoxicity may well represent part of the mechanism by which conceptus rejection takes place. This concept should favour administration of low glucocorticoid doses with a view to preventing sporadic and recurrent abortion, a philosophy currently applied in our clinical practice, which urgently requires extensive investigation. Indeed, it can be speculated that while progesterone is needed in order to prepare the endometrium for the earliest phase of implantation, the subsequent stages of placentation depend on adequate maternal glucocorticoid production, aimed at controlling the factors involved in the inflammatory response which occurs naturally in any tissue remodeling. Based on the above considerations, the administration of glucocorticoids in early pregnancy could be useful to induce the appropriate maternal cytokine environment. Such a role of corticosteroids in securing pregnancy from inflammatory wastage is further enforced by the evidence that mifepristone, a drug employed to induce medical abortion, is a potent antiglucocorticoid [108].

In light of the recent demonstration that some antibiotics, such as ampicillin, reduce amniotic PGE_2 _[109,110] and IL-6 release both *in vitro *and *in vivo *[111], by a mechanism independent of their antibacterial properties, their use in the management of the feto-maternal inflammatory state should also be considered. Based on the detrimental role of TXA_2_/PGI_2_-imbalance in the pathogenesis of preeclampsia, a further therapeutic strategy could be represented by the administration of specific prostanoid blockers, such as pirmagrel, a strong inhibitor of thromboxane production in normal and preeclamptic cytotrophoblast [112]. In addition, the clinical benefits of heparin and aspirin in cases of poor pregnancy outcome, such as antiphospholipid syndrome, are also recognized. The use of low doses of these drugs in the management of early pregnancy dysfunction is justified by their influence on EVT differentiation [113] and apoptosis [114]. Finally, considering that inflammation and oxidative stress are features of preeclampsia, a combination of low-dose aspirin and antioxidants like vitamins C and E has been advocated in management of the syndrome, although the possible benefit of such a therapy is still awaiting adequate clinical investigation [95].

## Concluding remarks

Advances in recent years have demonstrated that pregnancy outcome depends upon a profound dialogue between the decidua and trophoblast. Such a dialogue is sustained by CT differentiation into ST, as well as EVT, in order to invade maternal spiral arteries for optimal fetal oxygenation and nutrition. A pivotal role in this process has been ascribed to unique uterine wall NK cells, which are characterized by the ability to produce anti-inflammatory cytokines. It is worth noting that both maternal and paternal factors are responsible for NK cell acquisition of this ability, which appears to depend upon a shift of Th1 towards the Th2 phenotype. Indeed, although a weak inflammatory state is observed during physiological gestation, an exacerbation of this condition is certainly involved in the pathological events leading to pregnancy loss. A delicate balance between free radicals and antioxidants is also essential for normal gestation, since low doses of radicals are thought to be beneficial for both embryo development and por-inflammatory cytokine control, although their potentiated levels may represent a teratologic threat to fetal tissues and are detrimental for placenta formation. In light of these conclusion, anti-inflammatory and antioxidant therapies are currently evaluated in an attempt to contrast the adverse conditions which lead to pathological pregnancies. Administration of glucocorticoids before conception, or during pregnancy, to prepare and maintain the proper environment for implantation, placentation and fetal growth, appears to represent a new strategy in the battle against pregnancy loss, needing thorough clinical investigation.

Challenges for future research will be to obtain a complete picture of the factors which influence the mechanism of placentation and to identify the very early markers of pathological pregnancies. Progress in this field could allow to design new drugs able to easily reach the feto-maternal unit and suitable for controlling the cytokines involved in the physiological progression of pregnancy.
